# Neutropenia management with palbociclib in Japanese patients with advanced breast cancer

**DOI:** 10.1007/s12282-019-00970-7

**Published:** 2019-05-24

**Authors:** Norikazu Masuda, Hirofumi Mukai, Kenichi Inoue, Yoshiaki Rai, Shinji Ohno, Yuko Mori, Satoshi Hashigaki, Yasuaki Muramatsu, Yoshiko Umeyama, Hiroji Iwata, Masakuzu Toi

**Affiliations:** 1 Department of Surgery, Breast Oncology, National Hospital Organization Osaka National Hospital, 2-1-14, Hoenzaka, Chuou-ku, Osaka, 540-0006 Japan; 2 Department of Breast and Medical Oncology, National Cancer Center Hospital East, 6-5-1, Kashiwanoha, Kashiwa-shi, Chiba, 277-8577 Japan; 3 Division of Breast Oncology, Saitama Cancer Center, 780 Komuro Inamachi Kitaadachi-gun, Saitama, 362-0806 Japan; 4 Sagara Hospital, 3-28 Tenokuchi-cho, Kagoshima City, 892-0845 Japan; 5 Breast Oncology Center, The Cancer Institute Hospital of JFCR, 3-8-31, Ariake, Koto-ku, Tokyo, 135-8550 Japan; 6 Pfizer Japan Inc, Tokyo, Japan; 7 Department of Breast Oncology, Aichi Cancer Center Hospital, 1-1, Kanokoden, Chikusa-ku, Nagoya, Aichi 464-8681 Japan; 8 Kyoto University Graduate School of Medicine, 54 Kawaracho, Shogoin, Sakyo-ku, Kyoto, 606-8507 Japan

**Keywords:** Advanced breast cancer, Cyclin-dependent kinase, Japanese patients, Palbociclib, Neutropenia

## Abstract

**Background:**

The cyclin-dependent kinase 4/6 (CDK4/6) inhibitor palbociclib, in combination with endocrine therapy (ET), significantly prolonged progression-free survival in women with hormone receptor–positive, human epidermal growth factor receptor 2–negative advanced breast cancer (HR+/HER2− ABC) in PALOMA-2 and PALOMA-3. Neutropenia and palbociclib dose reductions/interruptions occurred more frequently in the Japanese versus overall populations. We evaluated neutropenia patterns, palbociclib dose management, and clinical responses after dose reduction in Japanese patients in PALOMA-2 and PALOMA-3 and a single-arm Japanese phase 2 study.

**Methods:**

PALOMA-2 and the Japanese phase 2 study enrolled postmenopausal women with estrogen receptor–positive, HER2− ABC who had not received prior systemic therapy for advanced disease; PALOMA-3 enrolled women with HR+/HER2− ABC, regardless of menopausal status, whose disease had progressed after prior ET. Palbociclib (125 mg/day) was administered 3 weeks on/1 week off. Dose reduction/interruption, cycle delay, tumor response, and laboratory-assessed neutropenia were analyzed in Japanese patients who received palbociclib.

**Results:**

A total of 101 Japanese patients received palbociclib + ET. Among Japanese patients in the 3 studies, the frequency of all-grade/grade 3/grade 4 neutropenia was 94%/53%/34%, 100%/69%/21%, and 100%/67%/26%, respectively. Twenty (63%), 28 (67%), and 15 (56%) patients required palbociclib dose reduction. Dose interruption or reduction did not affect palbociclib treatment duration, and durable tumor response was observed despite dose reduction.

**Conclusion:**

Neutropenia was manageable with dose modifications, without affecting palbociclib treatment duration or efficacy.

**Trial registration:**

Pfizer (NCT01740427, NCT01684215, NCT01942135).

**Electronic supplementary material:**

The online version of this article (10.1007/s12282-019-00970-7) contains supplementary material, which is available to authorized users.

## Introduction

Breast cancer is the most frequently diagnosed cancer in women in Japan and worldwide [1, 2]. Although incidence rates have historically been highest in Western countries, in recent decades both incidence and mortality rates have increased in Asian countries, including Japan [1, 3–5]. The most common subtype of advanced breast cancer is hormone receptor–positive, human epidermal growth factor receptor 2–negative advanced breast cancer (HR+/HER2− ABC) [6]. For patients with HR+/HER2− ABC, treatment guidelines recommend the use of endocrine therapy (ET) in combination with a cyclin-dependent kinase 4/6 (CDK4/6) inhibitor [7, 8]. The CDK4/6 inhibitor palbociclib has demonstrated anticancer activity in preclinical assays [9, 10] as well as in phase 2 and 3 clinical trials of HR+/HER2− ABC [11–13]. In 2017, palbociclib was approved in Japan for the treatment of HR+/HER2− inoperable or recurrent breast cancer [14].

The safety and efficacy of palbociclib as treatment for women with HR+/HER2− ABC were investigated in 2 phase 3, randomized, double-blind, placebo-controlled, multicenter trials [12, 13]. Palbociclib was tested in combination with letrozole in the first-line setting in the PALOMA-2 study and in combination with fulvestrant in patients who progressed on prior ET in the PALOMA-3 study [12, 13, 15]. In both studies, palbociclib improved progression-free survival (PFS) in the overall population. Subgroup analyses of Asian and Japanese patients enrolled in PALOMA-2 and PALOMA-3 indicated that palbociclib is also safe and effective in this patient population [16–19]. A phase 2, single-arm, open-label study of palbociclib in combination with letrozole as first-line treatment was conducted in Japanese patients with HR+/HER2− ABC, and the combination treatment was found to be effective, with a manageable safety profile [20].

Differences in safety and dosing parameters were observed between Japanese patients and the overall population in the 2 phase 3 studies [17, 18]. The reported rate of neutropenia was higher in Japanese patients compared with the overall population. In the palbociclib arm of PALOMA-2 (data cutoff: February 26, 2016), all-grade neutropenia was reported in 94% and 80% of Japanese patients and the overall population, respectively [12, 17]. In the palbociclib arm of PALOMA-3 (data cutoff: December 5, 2014), 93% of Japanese patients reported all-grade neutropenia compared with 79% of the overall population [13, 18]. The incidence of febrile neutropenia was rare in both Japanese and overall populations in each study.

In PALOMA-2 and PALOMA-3, dosing schedule changes such as palbociclib dose reductions, interruptions, or cycle delays occurred more frequently in Japanese patients than in the overall populations. In the palbociclib arm of PALOMA-2, dose reductions and interruptions occurred in 63% and 69%, respectively, of Japanese patients compared to 36% and 67% of the overall population. In the palbociclib arm of PALOMA-3, 52% and 100% of Japanese patients experienced dose reductions and interruptions, respectively, compared to 32% and 87% of the overall population [17, 18].

To date, it is not clear how the differences in neutropenia patterns and dosing affect palbociclib treatment efficacy and duration in Japanese patients. This study reports our evaluation of neutropenia patterns, palbociclib dose management, and clinical response after dose reduction in Japanese patients enrolled in PALOMA-2, PALOMA-3, and the Japanese phase 2 clinical trial (NCT01740427, NCT01942135, NCT01684215).

## Patients and methods

### Study design and patients

Detailed methods for the component studies, including key inclusion and exclusion criteria, have been previously published [12, 13, 15, 20]. Study sites and investigators that participated in PALOMA-2, the Japanese phase 2 study, or PALOMA-3 are listed in Supplementary Table 1. Study designs for all 3 trials are presented in Supplementary Figure 1. In brief, PALOMA-2 was a double-blind, randomized, placebo-controlled, international, multicenter, phase 3 trial that enrolled postmenopausal women aged ≥ 18 years with estrogen receptor–positive (ER+), HER2− ABC who had not received prior systemic anticancer therapy for advanced disease [12]. The Japanese phase 2 study was a single-arm, open-label, multicenter trial in postmenopausal Japanese women aged ≥ 20 years with ER+/HER2− ABC who had not received prior systemic anticancer therapy for advanced disease [20]. PALOMA-3 was a double-blind, placebo-controlled, randomized, international, multicenter, phase 3 trial that enrolled female patients aged ≥ 18 years, regardless of menopausal status, with HR+/HER2− ABC whose disease had progressed on prior ET, that is, progression during or within 1 month of the end of prior ET in the context of advanced disease, or progression during or within 12 months after completion of adjuvant ET [13, 15].

### Treatment

In PALOMA-2, patients were randomized (2:1) to receive 125 mg palbociclib orally (PO) once daily for 3 weeks, followed by 1 week off treatment (termed “3/1 schedule” hereafter) or placebo plus letrozole [2.5 mg/day, PO; continuous] in 4-week cycles [12]. In the Japanese phase 2 study, all patients received palbociclib (125 mg/day, PO; 3/1 schedule) plus letrozole (2.5 mg/day, PO; continuous) in 4-week cycles [20]. In PALOMA-3, patients were randomized (2:1) to receive palbociclib (125 mg/day, PO; 3/1 schedule) or placebo plus fulvestrant (500 mg, intramuscular; every 14 days for 3 injections, then  every 28 days [q28d]) in 4-week cycles [13, 15]. The pre/perimenopausal patients also received goserelin administered subcutaneously q28d for the duration of the study [13, 15]. For all 3 studies, palbociclib dose modification (i.e., dose reduction/interruption or cycle delay) was permitted in the event of treatment-related toxicity, and the lowest palbociclib dose administered was 75 mg/day (Supplementary Table 2).

### Outcomes and assessments

Data from PALOMA-2 used in this analysis were based on a cutoff date of February 26, 2016 (accrual period, February 2013–July 2014). The data cutoff dates for the Japanese phase 2 study (accrual period, June 2014–February 2015) were March 4, 2016 (safety and laboratory data) and October 31, 2016 (efficacy and dosing data), and the data cutoff dates for PALOMA-3 (accrual period, October 2013–August 2014) were December 5, 2014 (safety and laboratory data) and October 23, 2015 (efficacy and dosing data). Tumor assessment, based on Response Evaluation Criteria in Solid Tumors v1.1, was performed by investigators at screening and every 12 weeks in PALOMA-2 and the Japanese phase 2 study, or every 8 weeks for the 1st year and every 12 weeks thereafter in PALOMA-3. Neutropenia assessments were based on laboratory data and not on reported adverse events. Laboratory tests were performed on days 1 and 15 of cycles 1 and 2, day 1 of subsequent cycles, and at the end of treatment. For pharmacokinetic analysis, blood samples were collected on day 15 of cycles 1 and 2 before study drug administration to determine the steady-state trough concentration (*C*_trough_) of palbociclib.

### Statistical analysis

All data were analyzed descriptively, except for the correlation between baseline neutrophil counts and posttreatment neutrophil counts. Pearson correlation coefficients were calculated for the analysis evaluating the correlation between baseline neutrophil counts and posttreatment neutrophil counts.

## Results

### Patient population

A total of 101 Japanese patients from the 3 studies were included in this analysis. Baseline demographics and disease characteristics of Japanese patients in the palbociclib arm of each study are presented in Table 1. Baseline demographics were generally similar across studies. Japanese patients in PALOMA-3 were approximately 10 years younger (median age, 53 years) than those in PALOMA-2 and the Japanese phase 2 study (median age, 67 and 63 years, respectively). Approximately half of the Japanese patients in PALOMA-3 were pre/perimenopausal. A higher percentage of patients in PALOMA-2 and PALOMA-3 had visceral metastases compared with patients in the Japanese phase 2 study (63%, 63%, and 48%, respectively). Median body weight of Japanese patients (53.0 kg) was lower than that of the overall population in PALOMA-2 (68.0 kg in the palbociclib–letrozole arm) and PALOMA-3 (67.2 kg in the palbociclib–fulvestrant arm). Similarly, the median body mass index of Japanese patients was lower than that of the overall population in PALOMA-2 (22.4 kg/m^2^ and 26.5 kg/m^2^, respectively) and in PALOMA-3 (22.1 kg/m^2^ and 26.1 kg/m^2^, respectively).

**Table 1:** Patient demographics and baseline disease characteristics

Characteristics	PAL + LET		PAL + FUL	Total (*N* = 101)
	PALOMA-2 (*n* = 32)	Japanese phase 2 (*n* = 42)	PALOMA-3 (*n* = 27)	
Age, median (range), year	67 (44–88)	63 (43–84)	53 (36‒77)	62 (36–88)
Weight, median (range), kg	54 (33–88)	50 (39–75)	54 (41‒83)	53 (33–88)
ECOG performance status, *n* (%)				
0	27 (84)	39 (93)	27 (100)	93 (92)
1	3 (9)	3 (7)	0	6 (6)
2	2 (6)	0	0	2 (2)
Menopausal status, *n* (%)				
Pre-/perimenopausal	–	–	13 (48)	13 (13)
Postmenopausal	32 (100)	42 (100)	14 (52)	88 (87)
Visceral metastases^a^, *n* (%)				
Yes	20 (63)	20 (48)	17 (63)	57 (56)
No	12 (38)	22 (52)	10 (37)	44 (44)
Measurable disease, *n* (%)				
Yes	28 (88)	36 (86)	21 (78)	85 (84)
No	4 (13)	6 (14)	6 (22)	16 (16)
Number of involved disease sites, *n* (%)				
1	7 (22)	12 (29)	7 (26)	26 (26)
2	10 (31)	13 (31)	12 (44)	35 (35)
3	12 (38)	12 (29)	4 (15)	28 (28)
4	3 (9)	2 (5)	4 (15)	9 (9)
> 4	0	3 (7)	0	3 (3)
Prior systemic therapies^b^				
Regimens, *n* (%)				
1	14 (44)	8 (19)	7 (26)	29 (29)
2	4 (13)	8 (19)	9 (33)	21 (21)
3	4 (13)	9 (21)	5 (19)	18 (18)
> 3	2 (6)	3 (7)	6 (22)	11 (11)
Prior systemic therapies^b^, *n* (%)				
Hormone therapy	21 (66)	27 (64)	27 (100)	75 (74)
Chemotherapy	15 (47)	20 (48)	15 (56)	50 (50)
Chemotherapy for advanced/metastatic	–	–	2 (7)	2 (2)
Prior lines of therapy in the context of metastatic disease				
Regimens, *n* (%)				
0	32 (100)	42 (100)	7 (26)^c^	81 (80)
1	–	–	12 (44)	12 (12)
2	–	–	5 (19)	5 (5)
≥ 3	–	–	3 (11)	3 (3)

*ECOG* Eastern Cooperative Oncology Group, *FUL* fulvestrant, *LET* letrozole, *PAL* palbociclib

^a^Refers to lung (including pleura) and/or liver involvement in Japanese phase 2 study and PALOMA-2 and refers to lung, liver, brain, pleural, or peritoneal involvement in PALOMA-3

^b^Prior systemic therapy was defined as any systemic therapy any time before study entry, including (neo)adjuvant therapy

^c^Patients who progressed on or ≤ 12 months after end of adjuvant therapy

### Dose level and treatment duration

Duration of treatment, dose reductions and interruptions, and relative dose intensities for Japanese patients in PALOMA-2, PALOMA-3, and the Japanese phase 2 study are presented in Supplementary Table 3. The proportion of Japanese patients who experienced dose reductions was relatively similar across studies, ranging from 56% in PALOMA-3 to 67% in the Japanese phase 2 study. All Japanese patients in PALOMA-3 experienced dose interruption compared with 69% and 86% in PALOMA-2 and the Japanese phase 2 study, respectively. Median relative dose intensity was highest in Japanese patients in PALOMA-3 and relatively similar in PALOMA-2 and the Japanese phase 2 study.

**Fig. 1:**
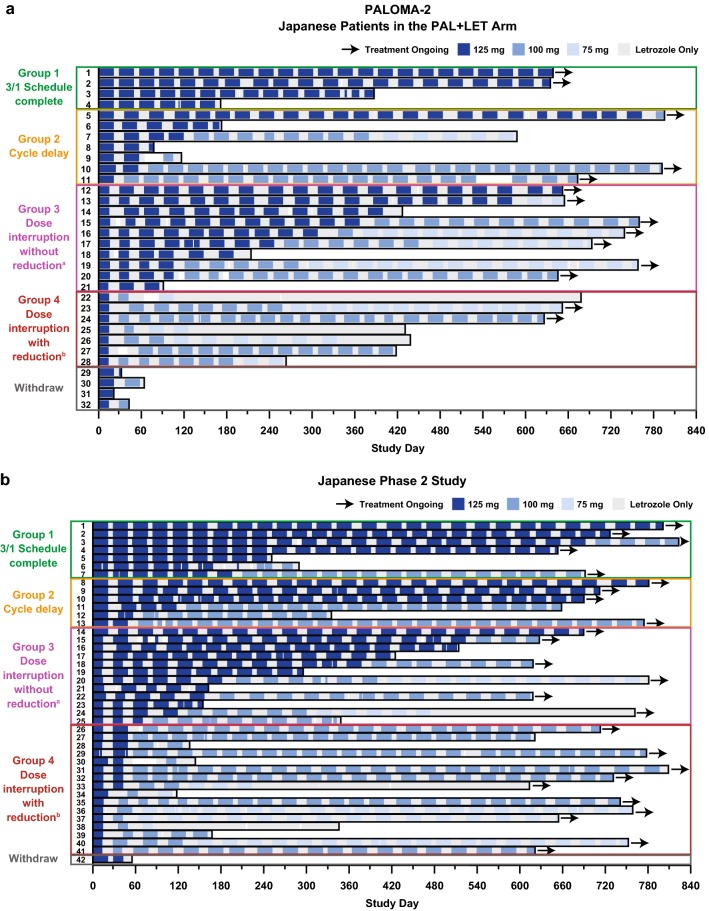

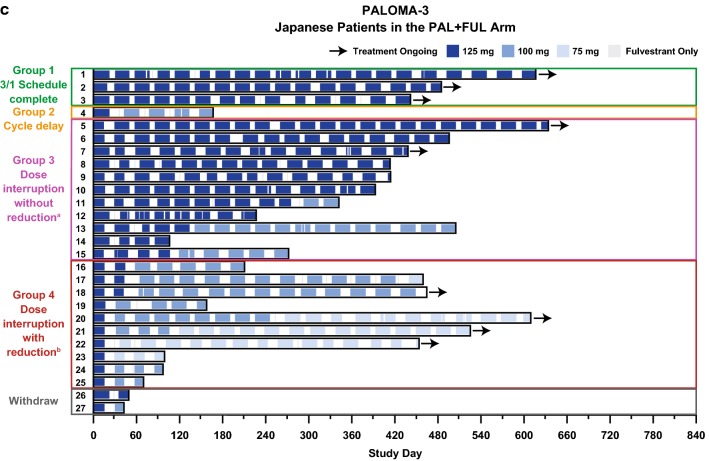
Dosing schedules in Japanese patients. Dosing schedules in Japanese patients who completed the 3/1 schedule, patients with cycle delay, and patients with dose interruption with and without dose reduction during the first 2 cycles in **a** PALOMA-2, **b** Japanese phase 2 study, and **c** PALOMA-3. *FUL* fulvestrant, *LET* letrozole, *PAL* palbociclib. ^a^Patients with interrupted palbociclib dose and no dose reduction during the first 2 cycles and/or at the start of cycle 3. ^b^Patients with interrupted palbociclib dose and dose reduction during the first 2 cycles and/or at the start of cycle 3. See Supplementary Figure 2 for detail regarding group categorization and dosing schedule examples

Japanese patients from each of the 3 studies were categorized into 4 groups based on their dosing schedule during the first 2 cycles (Supplementary Figure 2). The first group comprised those patients who completed the 3/1 schedule (i.e., 3 weeks of daily palbociclib and 1 week without palbociclib, comprising one 4-week cycle) without any palbociclib dose modifications during the first 2 cycles (Group 1). The second group comprised patients who experienced cycle delay without dose interruption at some point during the first 2 cycles (Group 2). The third group comprised patients who experienced palbociclib dose interruption at some point during the first 2 cycles but who did not require palbociclib dose reduction during the first 2 cycles and/or at the start of cycle 3 (Group 3). The fourth group comprised those patients who required both palbociclib dose interruption at some point during the first 2 cycles and dose reduction during the first 2 cycles and/or at the start of cycle 3 (Group 4) (Fig. 1a-c). Although the percentage of Japanese patients who completed the 3/1 schedule of palbociclib treatment during the first 2 cycles (Group 1) was small (12.5%, 16.7%, and 11.1% in PALOMA-2, the Japanese phase 2 study, and PALOMA-3, respectively), these patients generally continued treatment without dose reduction, except for 3 patients in the Japanese phase 2 study. Although the majority of Japanese patients in each study experienced cycle delay, dose interruption, or dose reduction during the first 2 cycles, these modifications of the palbociclib treatment schedule did not appear to affect treatment duration for individual patients in any study. The median duration of palbociclib treatment in Japanese patients who completed the 3/1 schedule (Group 1), had cycle delay (Group 2), had palbociclib dose interruption but no dose reduction (Group 3), and had palbociclib dose interruption and reduction (Group 4) was 511.0, 589.0, 653.5, and 439.0 days, respectively, in PALOMA-2; 693.0, 702.5, 567.5, and 639.5 days, respectively, in the Japanese phase 2 study; and 484.0, 167.0, 413.0, and 332.0 days in PALOMA-3 (Supplementary Table 4). In PALOMA-3, although median treatment duration in Japanese patients with both dose interruption and reduction (Group 4) was numerically shorter compared with that in other groups (Supplementary Table 4), some patients continued long-term treatment despite dose interruption and reduction (Fig. 1c).

**Fig. 2:**
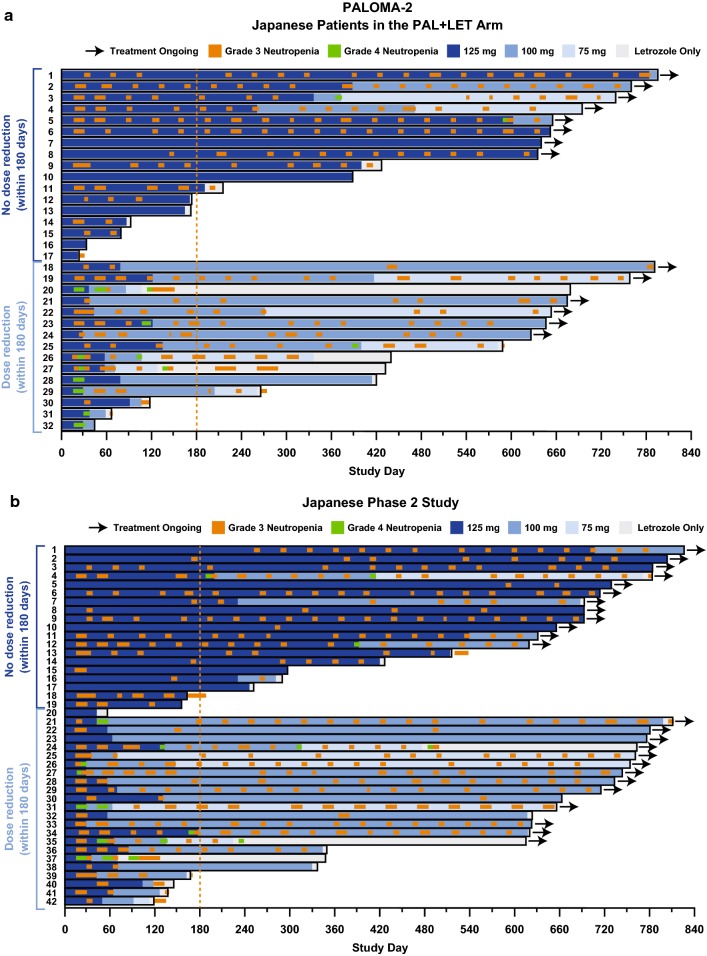

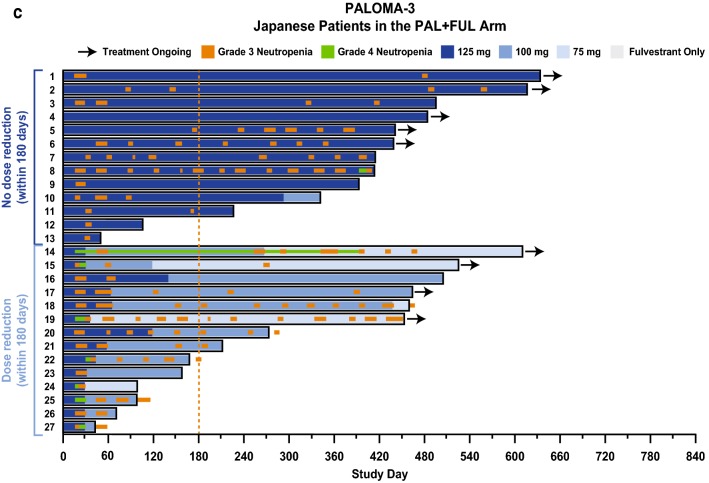
Dose levels and treatment duration in Japanese patients with and without dose reduction within 180 days of treatment initiation. **a** PALOMA-2, **b** Japanese phase 2 study, and **c** PALOMA-3. *FUL* fulvestrant, *LET* letrozole, *PAL* palbociclib

The effect of early palbociclib dose reduction (i.e., dose reduction within the first 180 days after treatment initiation) on treatment duration in Japanese patients was assessed in individual patients in each study (Fig. 2a–c). Within each study, Japanese patients were divided into 2 groups: those who experienced early dose reduction and those who did not. Median duration of treatment in Japanese patients with and without palbociclib dose reduction within 180 days of treatment initiation was 589.0 and 427.0 days, respectively, in PALOMA-2; 639.5 and 642.5 days, respectively, in the Japanese phase 2 study; and 241.5 and 413.0 days, respectively, in PALOMA-3 (Supplementary Table 4).

### Tumor response assessment

**Fig. 3:**
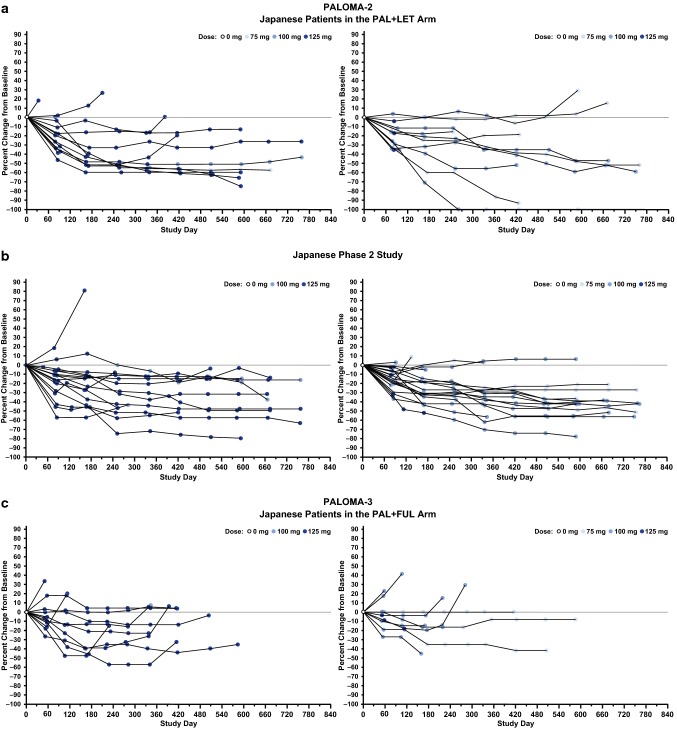
Tumor shrinkage in Japanese patients. Changes in tumor size for individual patients without (left) and with (right) dose reduction within 180 days of treatment initiation in **a** PALOMA-2, **b** Japanese phase 2 study, and **c** PALOMA-3. *FUL* fulvestrant, *LET* letrozole, *PAL* palbociclib

The effect of early palbociclib dose reduction on tumor response was also evaluated in Japanese patients. In all 3 studies, the percentage change in tumor size was relatively similar in patients who did and did not undergo dose reduction, suggesting that early dose reduction did not negatively impact tumor response (Fig. 3a–c; Supplementary Table 5). Most Japanese patients in PALOMA-2 and the Japanese phase 2 study experienced 30% or greater maximum reduction from baseline in tumor size [16 (64.0%) and 23 (63.9%) patients, respectively]. In both studies, the numbers of Japanese patients without and with early dose reduction who experienced 30% or greater maximum reduction were similar [PALOMA-2, 9 (64.3%) and 7 (63.6%) patients, respectively; Japanese phase 2, 10 (58.8%) and 13 (68.4%) patients, respectively]. In PALOMA-3, only 6 (28.6%) Japanese patients experienced 30% or greater tumor reduction from baseline; 4 (36.4%) of these had no early dose reduction and 2 (20.0%) had early dose reduction. Over the course of each study, tumor reduction occurred soon after treatment initiation and then stabilized; the reduction in tumor size in response to palbociclib treatment was generally maintained despite dose reduction (Fig. 3a–c).

### Neutropenia assessment

Almost all Japanese patients in the palbociclib arm of each study reported all-grade neutropenia (Table 2). Of these, approximately 90% of neutropenia occurrences were grade 3/4 in severity. After the first palbociclib dose, median time to onset of any-grade and grade 3/4 neutropenia was approximately 2 weeks in each study. The duration of all-grade neutropenia in Japanese patients was approximately 2 weeks and of grade 3/4 neutropenia was approximately 1 week across the studies. In all 3 studies, grade 3/4 neutropenia was manageable with dose interruption and/or dose reduction, and Japanese patients generally remained in the study (Fig. 2a–c). Japanese patients with grade 4 neutropenia required dose reduction but continued treatment in all 3 studies. Febrile neutropenia was reported in 2 Japanese patients: 1 patient in PALOMA-3, with an onset date of cycle 1 day 17 and a resolve date of cycle 1 day 17, and 1 patient in the Japanese phase 2 study, with an onset date of cycle 1 day 18 and a resolve date of cycle 2 day 1. Two patients in the Japanese phase 2 study were administered granulocyte colony-stimulating factor.

**Table 2:** Neutropenia frequency, onset, and duration in Japanese patients

	PAL + LET		PAL + FUL	Total (*N* = 101)
	PALOMA-2 (*n* = 32)	Japanese phase 2 (*n* = 42)	PALOMA-3 (*n* = 27)	
Frequency of neutropenia, *n* (%)^a^				
All grade	30 (94)	42 (100)	27 (100)	99 (98)
Grade 3	17 (53)	29 (69)	18 (67)	64 (63)
Grade 4	11 (34)	9 (21)	7 (26)	27 (27)
Time from first dose to first episode onset, median (range), day	15.0 (13–29)	15.0 (13–29)	15.0 (13–29)	15.0 (13–29)
Time from first dose to first grade 3 or higher episode onset, median (range), day	15.5 (13–143)	15.0 (13–280)	15.0 (14–82)	15.0 (13–280)
Duration of neutropenia by episode and grade^b^				
All grades, number of episodes	478	471	136	1085
All grades, median (range), day	14.0 (1–196)	14.0 (1–252)	14.0 (1–123)	14.0 (1–252)
Grade ≥ 3, number of episodes	248	262	69	579
Grade ≥ 3, median (range), day	7.0 (1–44)	7.0 (1–29)	8.0 (1–42)	7.0 (1–44)

*FUL* fulvestrant, *LET* letrozole, *PAL* palbociclib

^a^Neutropenia included laboratory tests of neutrophils (absolute or percentage)

^b^For duration of neutropenia by grade, all episodes are counted (e.g., if a patient had 3 episodes, then durations for all 3 episodes are added together and used in the calculation)

**Fig. 4:**
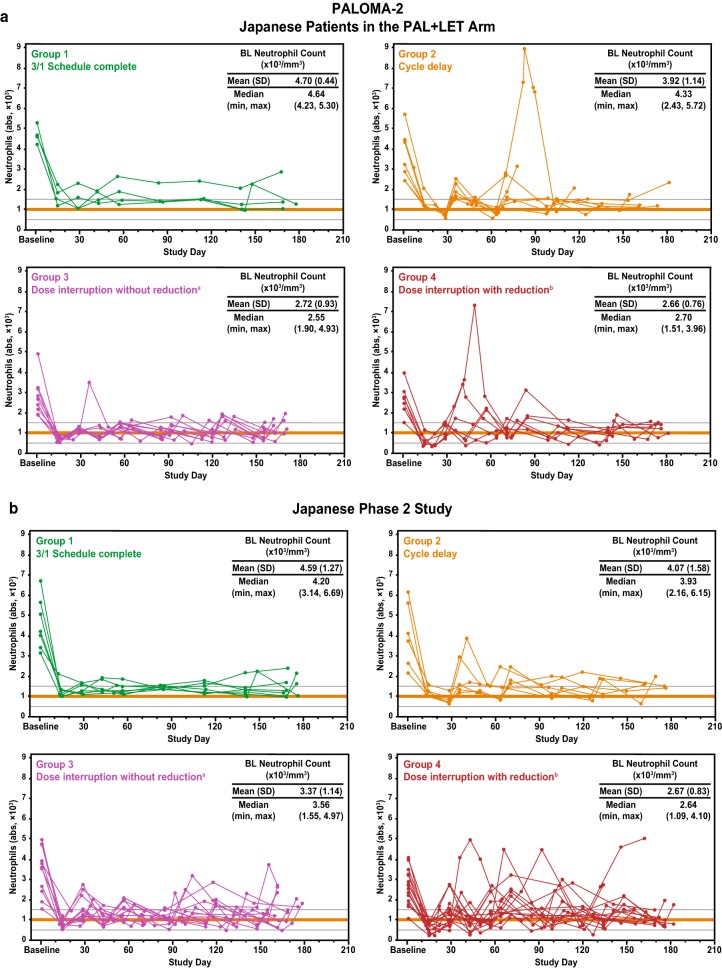

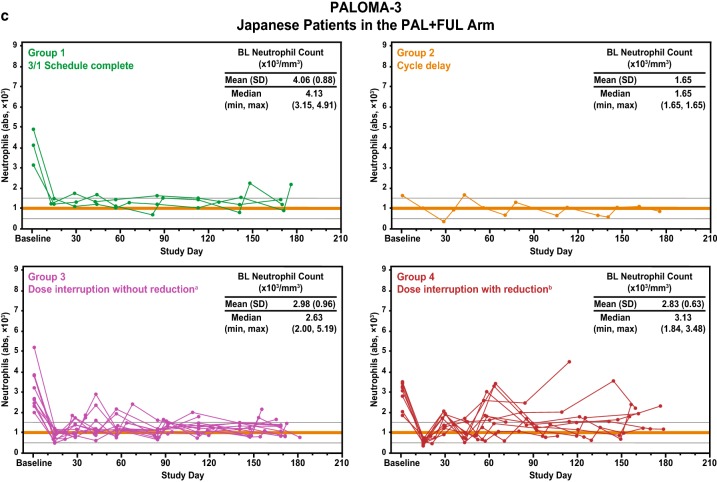
Time course of neutrophil count in Japanese patients. Neutrophil counts over time in patients who completed the 3/1 schedule, patients with cycle delay, and patients with dose interruption with and without dose reduction during the first 2 cycles in **a** PALOMA-2, **b** Japanese phase 2 study, and **c** PALOMA-3. *Abs* absolute, *BL* baseline, *FUL* fulvestrant, *LET* letrozole, *PAL* palbociclib. ^a^Patients with interrupted palbociclib dose and no dose reduction during the first 2 cycles and/or at the start of cycle 3; ^b^patients with interrupted palbociclib dose and dose reduction during the first 2 cycles and/or at the start of cycle 3. See Supplementary Figure 2 for detail regarding group categorization and dosing schedule examples

**Fig. 5:**
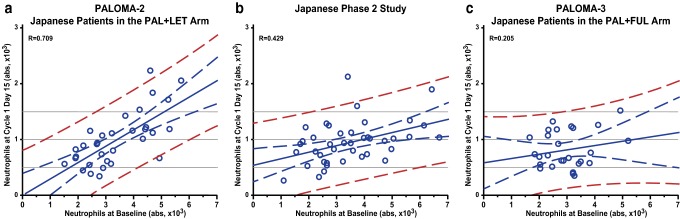
Correlation between baseline and cycle 1 day 15 neutrophil counts in Japanese patients. **a** PALOMA-2, **b** Japanese phase 2 study, and **c** PALOMA-3. *FUL* fulvestrant, *LET* letrozole, *PAL* palbociclib

Baseline neutrophil counts were analyzed within each patient group based on dosing schedules. In each study, Japanese patients who completed a 3/1 schedule (Group 1) had relatively higher neutrophil counts at baseline than did Japanese patients who required cycle delay or dose interruption (Groups 2, 3, and 4) (Fig. 4a-c). Japanese patients who required dose interruption (Groups 3 and 4) had relatively lower baseline neutrophil counts in all 3 studies. The correlation between baseline neutrophil levels and the neutrophil counts after treatment initiation in each study was investigated [16]. A positive correlation was observed between baseline neutrophil level and neutrophil count at cycle 1 day 15 in PALOMA-2 (*R* = 0.709) and the Japanese phase 2 study (*R* = 0.429) but not in PALOMA-3 (*R* = 0.205); this correlation was strongest in PALOMA-2 (Fig. 5a–c).

### Pharmacokinetics and dosing schedule

Individual palbociclib *C*_trough_ values at 125 mg dosing are presented by patient group based on dosing schedules in Supplementary Figure 3. In PALOMA-2 and in the Japanese phase 2 study, palbociclib *C*_trough_ in Japanese patients who required cycle delay or dose interruption (Groups 2, 3, and 4) tended to be slightly higher compared with Japanese patients who completed a 3/1 schedule (Group 1). However, in general the majority of individual palbociclib *C*_trough_ values in each of the 4 groups were within a similar range.

## Discussion

Analysis of the data from palbociclib-treated Japanese patients from the phase 3 PALOMA-2 and PALOMA-3 studies as well as a Japanese phase 2 study revealed that the frequency of neutropenia and palbociclib dose reductions, cycle delays, and dose interruptions was higher in Japanese patients than in the overall populations. However, these treatment modifications did not appear to have affected palbociclib treatment duration or tumor response in Japanese patients. Japanese patients who completed a 3/1 schedule during the first 2 cycles generally continued palbociclib treatment without dose reduction, and early dose reduction did not affect palbociclib treatment duration in Japanese patients.

Importantly, disease control was maintained in Japanese patients when the palbociclib dose was reduced. Durable objective response was observed after dose reduction in Japanese patients, a similar observation to that described in the overall populations in PALOMA-2 and PALOMA-3; exposure-response analyses of both studies revealed that patients who received palbociclib dose reductions exhibited similar PFS to patients without dose reductions [21, 22]. In Japanese patients, no apparent difference in tumor shrinkage was observed between patients with or without dose reduction, suggesting that reduction of palbociclib dose due to adverse events should have little impact on the efficacy of palbociclib.

The frequency of neutropenia was higher in Japanese patients than in the overall phase 3 clinical trial population; almost all of the Japanese patients reported any-grade neutropenia compared with approximately 80% of the overall populations. Grade 3/4 neutropenia was also more common in Japanese patients (approximately 90%) compared with the overall populations (approximately 65% in both PALOMA-2 and PALOMA-3). However, grade 3/4 neutropenia recovered promptly and was manageable in Japanese patients with dose interruption or reduction.

Japanese patients who completed a 3/1 schedule during the first 2 cycles had relatively higher baseline neutrophil counts; patients who required dose interruption had relatively lower baseline neutrophil counts. Decrease in neutrophil counts over time was not correlated with age, weight, body surface area, or body mass index in Japanese patients [17, 18]. These findings support those of a previous subgroup analysis of palbociclib efficacy and safety in Asian patients enrolled in the PALOMA-3 study, which suggested that neutropenia is not influenced by pharmacokinetics of palbociclib [16]. Neutrophil counts at cycle 1 day 15 did not correlate with palbociclib *C*_trough_ at cycle 1 day 15 in the overall population of PALOMA-2 and PALOMA-3 [17, 18]. Similarly, this correlation was not observed in the pooled Japanese patients from PALOMA-2, the Japanese phase 2 study or PALOMA-3 (data not shown). In addition, no apparent difference in palbociclib *C*_trough_ was observed by dosing schedules, although pharmacokinetic comparison among dosing groups was difficult due to the limited data available within each group. On the other hand, a correlation was observed between neutrophil counts on cycle 1 day 15 and baseline neutrophil levels in PALOMA-2 and the Japanese phase 2 study. Therefore, baseline neutrophil count could potentially serve as a predictor of the neutropenia pattern in the early treatment cycles.

In conclusion, although the frequency of neutropenia and palbociclib dose reductions, cycle delays, and dose interruptions was higher in Japanese patients than in the overall populations, neutropenia was manageable with dose modification, and these dose modifications were unlikely to have affected palbociclib treatment duration and efficacy in these patients. These findings suggest that it may be possible to predict a suitable treatment schedule for individual patients within early cycles, which would be useful in clinical practice.

## Electronic supplementary material

Below is the link to the electronic supplementary material.

Supplementary material 1 (DOCX 370 KB)
