# Supplementary material for: Neutropenia management with palbociclib in Japanese patients with advanced breast cancer
Source: Breast Cancer. 2019 May 24;26(5):637–50. doi: 10.1007/s12282-019-00970-7 (PMC6694088; doi:10.1007/s12282-019-00970-7)
Supplement: Supplementary file 1 — Supplementary material 1 (DOCX 370 KB) [file 12282_2019_970_MOESM1_ESM.docx]

**Electronic Supplementary Material**

**Neutropenia Management With Palbociclib in Japanese Patients With Advanced Breast Cancer**

*Breast Cancer*

Norikazu Masuda,* Hirofumi Mukai, Kenichi Inoue, Yoshiaki Rai, Shinji Ohno, Yuko Mori, Satoshi Hashigaki, Yasuaki Muramatsu, Yoshiko Umeyama, Hiroji Iwata, Masakuzu Toi

*Corresponding Author

Norikazu Masuda, MD, PhD

NHO Osaka National Hospital

Department of Surgery, Breast Oncology

Email: [nmasuda@alpha.ocn.ne.jp](mailto:nmasuda@alpha.ocn.ne.jp)

**Supplementary Information**

**Supplementary Figure 1. PALOMA-2, Japanese Phase 2 Study, and PALOMA-3 Study Designs**


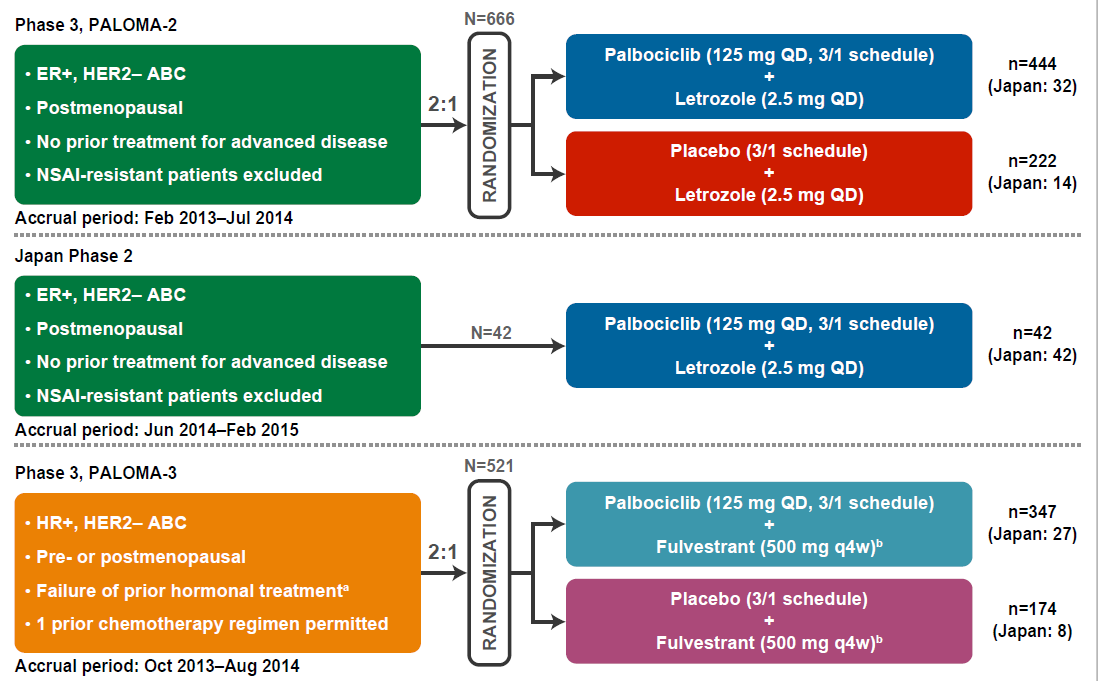


ABC=advanced breast cancer; AI=aromatase inhibitor; ER=estrogen receptor; HER2=human epidermal growth factor receptor 2; HR=hormone receptor; NSAI=nonsteroidal aromatase inhibitor; QD=once daily; q4w=every 4 weeks.

^a^Progressed on or ≤12 months after end of adjuvant therapy (AI or tamoxifen) or progressed on or ≤1 month after end of treatment with AI/other endocrine therapy for advanced disease.

^b^Fulvestrant 500 mg was administered intramuscularly on days 1 and 15 of cycle 1 and every 28 days (+/- 7 days) thereafter starting from day 1 of cycle 1.

**Supplementary Figure 2. Examples of Palbociclib Dosing Schedules for Each Group**


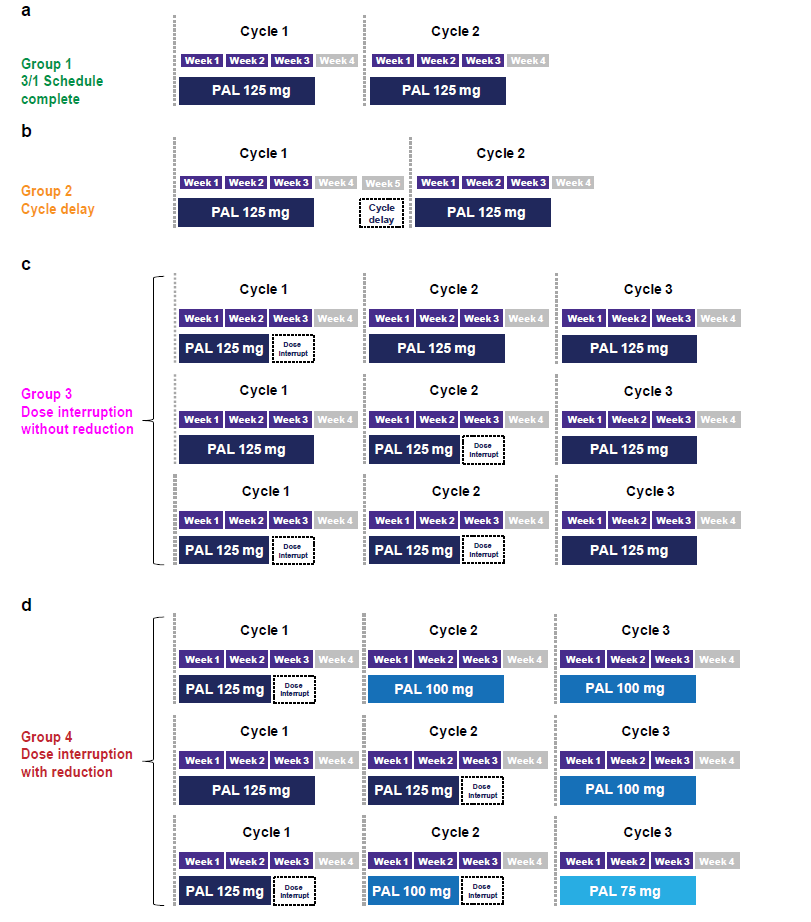


(a) Group 1 includes patients who completed the 3/1 schedule (ie, 3 weeks of daily palbociclib and 1 week without palbociclib, comprising one 4-week cycle) without any palbociclib dose modifications during the first 2 cycles.

(b) Group 2 includes patients who experienced cycle delay without dose interruption at some point during the first 2 cycles.

(c) Group 3 includes patients who experienced palbociclib dose interruption at some point, regardless of cycle delay, during the first 2 cycles but who did not require palbociclib dose reduction during the first 2 cycles and/or at the start of cycle 3. For example, patients who experienced dose interruption at cycle 1 or cycle 2 or both cycles.

(d) Group 4 includes patients who required both palbociclib dose interruption at some point, regardless of cycle delay, during the first 2 cycles and dose reduction during the first 2 cycles and/or at the start of cycle 3. For example, patients who experienced dose interruption at cycle 1 or cycle 2 or both cycles and experienced dose reduction during cycle 2, at the start of cycle 3, or both.

**Supplementary Figure 3. Palbociclib *C_trough_* Values at Cycle 1 Day 15 by Dosing Schedules in Japanese Patients With 125 mg Dosing**


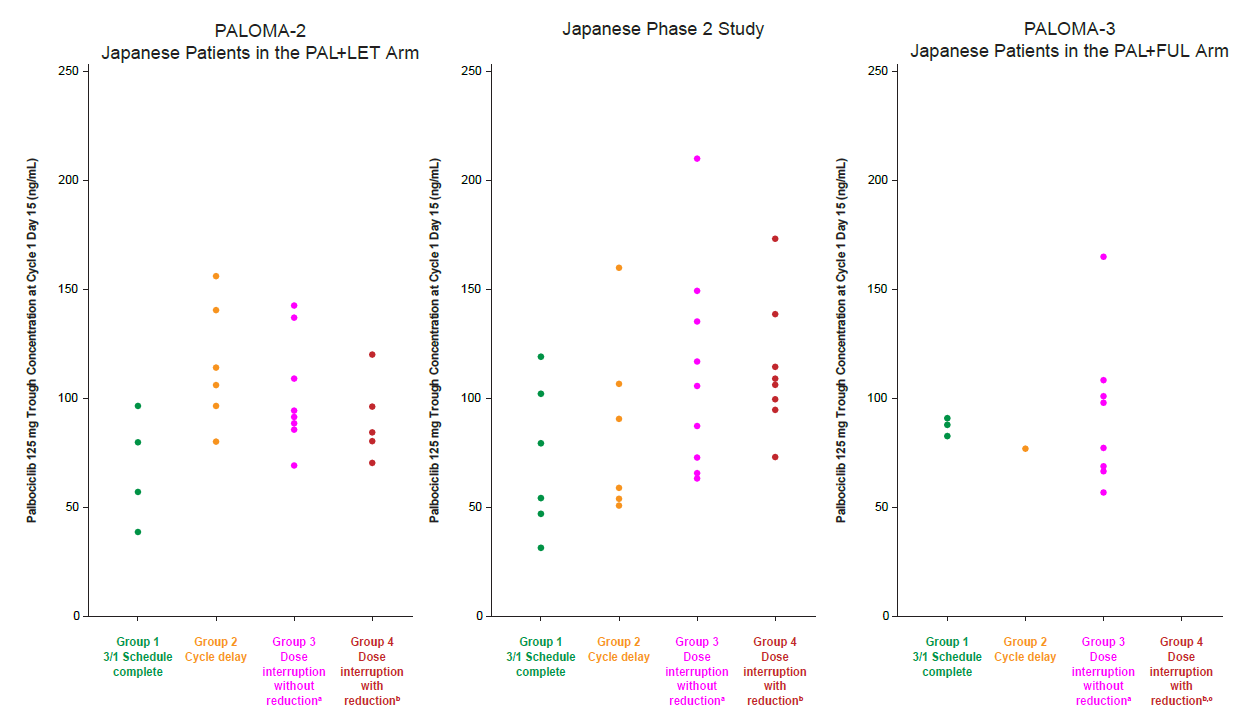


*C_trough_*= trough concentration; FUL=fulvestrant; LET=letrozole; PAL=palbociclib.

^a^Patients with interrupted palbociclib dose and no dose reduction during the first 2 cycles and/or at the start of cycle 3. See Supplementary Figure 2 for detail regarding group categorization and dosing schedule examples.

^b^Patients with interrupted palbociclib dose and dose reduction during the first 2 cycles and/or at the start of cycle 3. See Supplementary Figure 2 for detail regarding group categorization and dosing schedule examples.

^c^No pharmacokinetic data were available for patients with “Dose interruption with reduction” in PALOMA-3.

**Supplementary Table 1. List of Japanese Sites and Investigators Who Participated in PALOMA-2, Japanese Phase 2 study, or PALOMA-3**

| Site | Principal Investigator |
| --- | --- |
| Kyoto University Graduate School of Medicine | Masakazu Toi,^a^ Hiroshi Ishiguro, Megumi Takeuchi |
| Aichi Cancer Center Hospital | Hiroji Iwata^b^ |
| National Cancer Center Hospital | Kenji Tamura, Chikako Shimizu |
| National Cancer Center Hospital East | Hirofumi Mukai |
| National Hospital Organization Osaka National Hospital | Norikazu Masuda |
| Hakuaikai Medical Corporation Sagara Hospital | Yoshiaki Rai, Yasuaki Sagara |
| Iwate Medical University Hospital | Masahiro Kashiwaba, Kazushige Ishida |
| National Hospital Organization Shikoku Cancer Center | Shozo Ohsumi, Fumikata Hara |
| National Hospital Organization Kyushu Cancer Center | Shinji Ohno, Mayumi Ishida, Eriko Tokunaga |
| National Hospital Organization Hokkaido Cancer Center | Masato Takahashi |
| Chiba Cancer Center | Naohito Yamamoto, Rikiya Nakamura |
| Hiroshima City Hiroshima Citizens Hospital | Kenji Higaki, Shoichiro Ohtani |
| Kumamoto University Hospital | Hirotaka Iwase |
| Saitama Cancer Center | Kenichi Inoue |
| Kumamoto City Hospital | Reiki Nishimura, Yasuhiro Okumura |
| Kumamoto Shinto General Hospital | Reiki Nishimura |
| Niigata Cancer Center Hospital | Nobuaki Sato |

^a^Steering committee member of PALOMA-2.

^b^Steering committee member of PALOMA-3.

**Supplementary Table 2. Palbociclib Treatment and Dose Modification**

| Toxicity | PALOMA-2  Japan Phase 2 | PALOMA-3 |
| --- | --- | --- |
| Uncomplicated grade 3 neutropenia | Same dose | Same dose or ↓1 dose^a^ |
| Grade 3 neutropenia associated with a documented infection or fever ≥38.5°C | ↓1 dose | ↓1 dose or ↓2 doses^a^ |
| Grade 4 neutropenia | ↓1 dose | ↓1 dose or ↓2 doses^b^ |
| Grade 4 thrombocytopenia^c^ | ↓1 dose | ↓1 dose or ↓2 doses^d^ |
| Grade ≥3 nonhematologic toxicity  (only if persisting despite optimal medical treatment) | ↓1 dose | ↓1 dose or ↓2 doses^e^ |

^a^If neutrophil recovery is delayed beyond 7 days.

^b^In case of recurrent grade 4 event.

^c^In PALOMA-3, grade 3 or 4 thrombocytopenia.

^d^In case of recurrent grade ≥3 event.

^e^If repeated toxicity is seen in the next cycle or if recovery from grade 3 is delayed beyond 7 days.

**Supplementary Table 3. Exposure to Palbociclib**

|  | **PAL+LET** | | **PAL+FUL** |
| --- | --- | --- | --- |
|  | **PALOMA-2**  **(n=32)** | **Japan Phase 2 (n=42)** | **PALOMA-3**  **(n=27)** |
| Duration of treatment,^a^ median (range), d | 406 (21–796) | 643 (56–826) | 413 (42–634) |
| Average daily dose, median (range), mg | 112 (84–125) | 102 (75–125) | 113 (78–125) |
| Dose reductions,^b^ n (%) | 20 (63) | 28 (67) | 15 (56) |
| Reduction to 100 mg | 11 (34) | 20 (48) | 10 (37) |
| Reduction to 75 mg | 9 (28) | 8 (19) | 3 (11) |
| Reduction to 75 mg 2 weeks on/2 weeks off^c^ | N/A | N/A | 2 (7) |
| Time to first dose reduction,^d^ median (range), d | 63 (29–785) | 65.5 (29–708) | 36 (29–293) |
| Dose interruption,^e^ n (%) | 22 (69) | 36 (86) | 27 (100) |
| Relative dose intensity, median (range), % | 74 (44–100) | 73 (38–100) | 83 (51–99) |

d=day; FUL=fulvestrant; LET=letrozole; N/A=not applicable; PAL=palbociclib.

^a^Total number of days from the first, up to and including last day of each study treatment.

^b^Includes any dose reduction from initial prescribed dose; does not include dose interruptions. No changes in letrozole dose were allowed. All patients with a dose reduction were first reduced to 100 mg.

^c^Palbociclib 75 mg/d 2 weeks on followed by 2 weeks off, only allowed in PALOMA-3.

^d^Timed from start date of first occurrence minus first dose date of cycle 1 + 1.

^e^Interruptions include missed dose based on the case report form and dose administered = 0 mg.

**Supplementary Table 4. Treatment Duration by Palbociclib Dose Modification in Japanese Patients**

| Study | Category^a^ | Duration of Treatment (Days) | | | | | | |
| --- | --- | --- | --- | --- | --- | --- | --- | --- |
|  |  | **N (%)** | **Mean** | **SD** | **Median** | **Min** | **Max** |  |
| PALOMA-2  (Japanese Patients in the PAL+LET Arm) | All^b^ | 32 (100) | 448.8 | 269.9 | 514.0 | 21.0 | 796.0 |  |
|  | Groups |  |  |  |  |  |  |  |
|  | 3/1 schedule complete (Group 1) | 4 (12.5) | 458.3 | 225.0 | 511.0 | 171.0 | 640.0 |  |
|  | Cycle delay (Group 2) | 7 (21.9) | 459.7 | 324.6 | 589.0 | 77.0 | 796.0 |  |
|  | Dose interruption without reduction (Group 3)^c^ | 10 (31.3) | 563.8 | 238.2 | 653.5 | 92.0 | 760.0 |  |
|  | Dose interruption with reduction (Group 4)^d^ | 7 (21.9) | 501.9 | 153.9 | 439.0 | 264.0 | 679.0 |  |
|  | No dose reduction (within 180 days) | 17 (53.1) | 421.5 | 289.3 | 427.0 | 21.0 | 796.0 |  |
|  | Dose reduction (within 180 days) | 15 (46.9) | 479.9 | 252.5 | 589.0 | 43.0 | 792.0 |  |
| Japanese Phase 2 Study | All^b^ | 42 (100) | 547.8 | 242.8 | 642.5 | 56.0 | 826.0 |  |
|  | Groups |  |  |  |  |  |  |  |
|  | 3/1 schedule complete (Group 1) | 7 (16.7) | 606.7 | 237.1 | 693.0 | 252.0 | 826.0 |  |
|  | Cycle delay (Group 2) | 6 (14.3) | 660.2 | 165.8 | 702.5 | 336.0 | 783.0 |  |
|  | Dose interruption without reduction (Group 3)^c^ | 12 (28.6) | 500.9 | 220.4 | 567.5 | 155.0 | 783.0 |  |
|  | Dose interruption with reduction (Group 4)^d^ | 16 (38.1) | 545.7 | 263.2 | 639.5 | 119.0 | 811.0 |  |
|  | No dose reduction (within 180 days) | 20 (47.6) | 538.5 | 248.7 | 642.5 | 56.0 | 826.0 |  |
|  | Dose reduction (within 180 days) | 22 (52.4) | 556.2 | 242.8 | 639.5 | 119.0 | 811.0 |  |
| PALOMA-3  (Japanese Patients in the PAL+FUL Arm) | All^b^ | 27 (100) | 339.9 | 189.6 | 413.0 | 42.0 | 634.0 |  |
|  | Groups |  |  |  |  |  |  |  |
|  | 3/1 schedule complete (Group 1) | 3 (11.1) | 514.0 | 91.8 | 484.0 | 441.0 | 617.0 |  |
|  | Cycle delay (Group 2) | 1 (3.7) | 167.0 | – | 167.0 | 167.0 | 167.0 |  |
|  | Dose interruption without reduction (Group 3)^c^ | 11 (40.7) | 384.8 | 145.3 | 413.0 | 105.0 | 634.0 |  |
|  | Dose interruption with reduction (Group 4)^d^ | 10 (37.0) | 314.4 | 206.4 | 332.0 | 70.0 | 610.0 |  |
|  | No dose reduction (within 180 days) | 13 (48.1) | 388.3 | 174.1 | 413.0 | 49.0 | 634.0 |  |
|  | Dose reduction (within 180 days) | 14 (51.9) | 294.9 | 198.4 | 241.5 | 42.0 | 610.0 |  |

FUL=fulvestrant; LET=letrozole; PAL=palbociclib.

^a^Based on dosing schedule during the first 2 cycles.

^b^Includes the 4 Japanese patients in the PAL arm of PALOMA-2, 1 patient in the Japanese phase 2 study, and 2 Japanese patients in the PAL arm of PALOMA-3 who withdrew from the study.

^c^Patients with interrupted palbociclib dose and no dose reduction during the first 2 cycles and/or at the start of cycle 3.

^d^Patients with interrupted palbociclib dose and dose reduction during the first 2 cycles and/or at the start of cycle 3.

**Supplementary Table 5. Tumor Reduction From Baseline by Palbociclib Dose Reduction in Japanese Patients**

| **Study** | **Category** **by maximum % of tumor reduction, %** | **Dose Reduction Within 180 Days** | | | |
| --- | --- | --- | --- | --- | --- |
|  |  | **No** | | **Yes** | |
|  |  | **N** | **%** | **N** | **%** |
| **PALOMA-2**  **(Japanese patients in the PAL+LET arm)** | ≥30 | 9 | 64.3 | 7 | 63.6 |
|  | 20‒<30 | – | ­– | 1 | 9.1 |
|  | 10‒<20 | 2 | ­14.3 | 1 | 9.1 |
|  | 0‒<10 | 1 | 7.1 | 2 | 18.2 |
|  | <0 | 2 | 14.3 | – | ­– |
| **Japanese phase 2 study** | ≥30 | 10 | 58.8 | 13 | 68.4 |
|  | 20‒<30 | 1 | 5.9 | 2 | 10.5 |
|  | 10‒<20 | 5 | 29.4 | 1 | 5.3 |
|  | 0‒<10 | – | – | 2 | 10.5 |
|  | <0 | 1 | 5.9 | 1 | 5.3 |
| **PALOMA-3**  **(Japanese patients in the PAL+FUL arm)** | ≥30 | 4 | 36.4 | 2 | 20.0 |
|  | 20‒<30 | 1 | 9.1 | 1 | 10.0 |
|  | 10‒<20 | 3 | 27.3 | 2 | 20.0 |
|  | 0‒<10 | 1 | 9.1 | 3 | 30.0 |
|  | <0 | 2 | 18.2 | 2 | 20.0 |

FUL=fulvestrant; LET=letrozole; PAL=palbociclib.
